# Associations of smoking with overall obesity, and central obesity: a cross-sectional study from the Korea National Health and Nutrition Examination Survey (2010-2013)

**DOI:** 10.4178/epih.e2016020

**Published:** 2016-05-19

**Authors:** Yeonjung Kim, Seong Min Jeong, Bora Yoo, Bitna Oh, Hee-Cheol Kang

**Affiliations:** Department of Family Medicine, Severance Hospital, Yonsei University College of Medicine, Seoul, Korea

**Keywords:** Smoking, Obesity, Body mass index, Waist circumference, Risk factors, Cross-sectional studies

## Abstract

**OBJECTIVES:**

The association between smoking and obesity is a significant public health concern. Both are preventable risk factors of cardiovascular disease and a range of other conditions. However, despite numerous previous studies, no consensus has emerged regarding the effect of smoking on obesity. We therefore carried out a novel study evaluating the relationship between smoking and obesity.

**METHODS:**

A total of 5,254 subjects aged 19 years or older drawn from the 2010-2013 Korea National Health and Nutrition Examination Survey were included in this cross-sectional study. Smoking was examined both in terms of smoking status and the quantity of cigarettes smoked by current smokers. Multiple logistic regression analysis was used to assess the association between smoking and obesity. Overall obesity was defined as a body mass index (BMI) ≥25 kg/m^2^, and central obesity was defined as a waist circumference ≥90 cm for males and ≥85 cm for females. We adjusted for the possible confounding effects of age, sex, physical activity, alcohol consumption, and the presence of hypertension or diabetes.

**RESULTS:**

A statistically significant difference in central obesity according to smoking status was identified. Current smokers were more likely to be centrally obese than never-smokers (adjusted odds ratio,1.30; 95% confidence interval, 1.02 to 1.67). However, no significant association was found between smoking and obesity defined by BMI. Moreover, among current smokers, no statistically significant association was found between the daily amount of smoking and obesity or central obesity.

**CONCLUSIONS:**

Smoking was positively associated with central obesity. Current smokers should be acquainted that they may be more prone to central obesity.

## INTRODUCTION

Smoking and obesity are both crucial public health problems. Smoking increases the risk of cardiovascular diseases, cancer, and respiratory diseases [1]. Obesity is also associated with the incidence of many co-morbidities, such as type 2 diabetes, cancer, and cardiovascular diseases [2]. In addition, central obesity, defined in terms of waist circumference (WC), is known to be a better predictor of many obesity-related health problems, such as hypertension, diabetes, and cardiovascular diseases, than traditional obesity, which is defined according to body mass index (BMI) [3].

The association between smoking and obesity is a major public health concern because both are risk factors for cardiovascular disease and other conditions. However, no consensus exists regarding the relationship between smoking and obesity. In the World Health Organization Monitoring Trends and Determinants in Cardiovascular Disease (MONICA) Project, regular smokers generally had a lower BMI than never-smokers [4]. A cross-sectional study conducted in the United Kingdom also showed that current smokers were less likely to be obese than never-smokers [5]. However, other studies have found no significant association between BMI and smoking status [6], although some studies have reported smoking to be associated with central obesity instead of obesity defined in terms of BMI [6-8].

The purpose of this study was to characterize the overall association between smoking and obesity in the Korean general population using data from the Korea National Health and Nutrition Examination Survey (KNHANES).

## MATERIALS AND METHODS

### Study subjects

We conducted a cross-sectional study using data from the fifth (2010-2012) and part of the sixth (2013) KNHANES. The KNHANES is a representative epidemiological survey of the Korean population conducted by the Korean Ministry of Health and Welfare and the Korea Centers for Disease Control and Prevention. It is composed of three main surveys: a health interview, a health examination, and a nutrition survey. The information collected in the health interview included age, sex, physical activity measurements, alcohol consumption, and the presence of hypertension or diabetes, while the health examination provided data on anthropometric measures (height, weight, and WC). Between 2010 and 2013, 24,363 adults older than 19 years of age were surveyed. Of these, 210 participants who had missing values for anthropometric measurements were excluded. We excluded 1,084 subjects due to incomplete or missing data regarding their smoking status. Subjects were additionally excluded if any of the data for physical activity, alcohol consumption, and the presence of hypertension or diabetes were missing. This resulted in a final analytical sample of 5,254 subjects aged 19 years or older.

### Anthropometric measurements

Participants were asked to remove their shoes and heavy items of outer clothing before their weight, height, and WC were measured. Height was measured to the nearest 0.1 cm and weight was measured to the nearest 0.1 kg. WC was measured to the nearest 0.1 cm at the midpoint between the lower margin of the rib cage and the top of the iliac crest. BMI was derived from weight and height using the following formula: weight (kg)/(height [m]×height [m]). Obesity was defined as a BMI ≥25 kg/m^2^ [9]. Central obesity was defined as a WC ≥90 cm for males and ≥85 cm for females [9,10].

### Smoking behavior

Smoking behavior was assessed using a self-completed questionnaire. The information collected included the current smoking status and daily smoking amount of current smokers. Participants were categorized by smoking status as current, former, or never-smokers. The amount smoked by current smokers was assessed in terms of the number of cigarettes smoked per day, and current smokers were categorized as heavy smokers (>20 cigarettes per day), moderate smokers (11-20 cigarettes per day), and light smokers (≤10 cigarettes per day).

### Physical activity, alcohol consumption, presence of hypertension and diabetes

Physical activity, alcohol consumption, and the presence of hypertension and diabetes were self-reported through a questionnaire. Physical activity was categorized using the International Physical Activity Questionnaire Short Form (IPAQ-SF). The IPAQ-SF categorizes physical activity into three levels: (1) low, including individuals that do not meet the criteria for the other categories; (2) moderate, reflecting three or more days of vigorous activity for at least 20 minutes per day or five or more days of any combination of walking, moderate-intensity, or vigorous activities achieving a minimum of at least 600 metabolic equivalent of task (MET)-min/wk; and (3) high, corresponding to vigorous activity on at least three days, achieving a minimum of at least 1,500 MET-min/wk, or seven or more days of any combination of walking, moderate-intensity or vigorous activities, achieving a minimum of at least 3,000 MET-min/wk [11]. Alcohol intake was also self-reported as the frequency of alcohol consumption per week. We categorized alcohol consumption into four groups: less than once a week, one times per week, two to three times per week, and more than four times per week. The presence of hypertension and diabetes was assessed by self-reports of a diagnosis made by a physician.

### Statistical analysis

All statistical analyses were conducted using SPSS 18.0 (SPSS Inc., Chicago, IL, USA). For all analyses, two-tailed p-values <0.05 were considered to indicate statistical significance. The results were weighted to represent the Korean population, using weights calculated in order to account for the complex survey design, non-response rate, and post-stratification. The general characteristics of the never-smokers, current smokers, and former smokers were compared using analysis of variance (for continuous variables) or the chi-square test (for categorical variables). Multivariate logistic regression analysis was used to assess the associations of smoking status and amount with obesity and central obesity, adjusting for the potential confounding effects of age, sex, physical activity, alcohol consumption, hypertension, and diabetes. Analysis of covariance was used to explore the association of smoking status and amount with BMI and WC, adjusting for the potential confounding effects mentioned above.

## RESULTS

### General characteristics

The general characteristics of the study according to smoking status are presented in Table 1. Among the participants, 54.3% were never-smokers, 22.1% were current smokers, and 23.6% were former smokers. The average ages of never-smokers, current smokers, and former smokers were 44.64 years, 43.13 years, and 51.59 years, respectively. The BMI was highest in the current smokers and lowest in the never-smokers. WC was highest in the former smokers and lowest in the never-smokers. Statistically significant differences were found between smoking status and age, sex, alcohol intake, and physical activity (p<0.001).

### Smoking status

The mean BMI and WC values according to smoking status are shown in Table 2. Statistically significant differences in BMI were found among current smokers, former smokers, and never-smokers after adjusting for age, sex, alcohol consumption, physical activity, and the presence of hypertension and diabetes. The mean BMI value was highest among current smokers and lowest among former smokers after adjustment (p<0.05). Current smokers had the highest adjusted values of WC, and former smokers had the lowest WC values. However, no statistically significant correlation was found for WC.

The prevalence of obesity was highest among former smokers and lowest among never-smokers. Likewise, central obesity was most prevalent among former smokers and least prevalent among never-smokers (Table 3). In Table 4, multivariate regression analysis confirmed that current smokers were more likely to be obese than never-smokers (odds ratio [OR], 1.50; 95% confidence interval [CI], 1.27 to 1.77), and former smokers were also more likely to be obese than never-smokers (OR, 1.36; 95% CI, 1.16 to 1.61). These results were statistically significant (p<0.001). After adjusting for age, sex, levels of physical activity and alcohol consumption, and the presence of hypertension and diabetes, current smokers were more likely to be obese than never-smokers (OR, 1.14; 95% CI, 0.92 to 1.41), and former smokers were less likely to be obese than never-smokers (OR, 0.88; 95% CI, 0.70 to 1.10). However, these results were not statistically significant after adjustment.

Current smokers were more likely to be centrally obese than never-smokers (OR, 1.37; 95% CI, 1.14 to 1.65), and former smokers were also more likely to be centrally obese (OR, 1.37; 95% CI, 1.15 to 1.63). After adjustment, current smokers were more likely to be centrally obese than never-smokers (OR, 1.30; 95% CI, 1.02 to 1.67), and former smokers were less likely to be centrally obese than never-smokers (OR, 0.95; 95% CI, 0.74 to 1.22). These results were statistically significant (p<0.05). In addition to the results above, due to the low smoking rate among females in Korea, we assessed the association between smoking and obesity in each sex separately. Similarly to the results found among the general population, current smokers were more likely to be centrally obese than never-smokers among both males and females.

### Current smokers

The mean BMI and WC values according to daily smoking amount are shown in Appendix 1. Although statistically insignificant differences in BMI and WC were found among light, moderate, and heavy smokers after adjusting for age, sex, alcohol consumption, physical activity, and the presence of hypertension and diabetes, the mean values of BMI and WC increased as the smoking amount increased.

Obesity was most prevalent among heavy smokers (more than 20 cigarettes per day) and least prevalent among light smokers (less than 10 cigarettes per day). Central obesity was most prevalent among heavy smokers and least prevalent among never-smokers. However, neither obesity nor central obesity exhibited significant differences among light, moderate, and heavy smokers (Appendix 2).

Although the multivariate logistic regression analysis of the risk of both obesity and central obesity in relation to smoking amount did not show statistically significant correlations (Table 5), an increase in the OR for obesity and central obesity was observed in association with increasing levels of smoking. After adjusting for confounding factors (age, sex, physical activity, alcohol consumption, and the presence of hypertension and diabetes), heavy smokers had an adjusted OR of 1.45 (95% CI, 0.80 to 2.63) compared to never-smokers for obesity. The adjusted OR for central obesity in heavy smokers was 1.41 (95% CI, 0.79 to 2.53) compared to light smokers.

## DISCUSSION

Drawing on data from the KNHANES, we assessed the overall relationship of smoking status and smoking amount with obesity and central obesity. Overall, current smokers were more likely to be obese and centrally obese than never-smokers. When adjustments were made for age, sex, physical activity, alcohol consumption, hypertension, and diabetes, the association between smoking status and central obesity was attenuated, but remained significant. Current smokers had a higher risk for central obesity than never-smokers, even adjusting for these confounding factors.

The results of this study differ from those of previous studies that have found current smokers to be less likely to be obese than never-smokers [5,12,13]. Our study is consistent, however, with studies that have found no significant associations between smoking status and obesity [6]. Although some studies have reported that smoking status was not significantly associated with increased central obesity [6], our study is in accordance with previous studies that have found an association between smoking status and central obesity [7,8,14].

The association between smoking status and central obesity does not imply causation. However, reversibility and a dose-response relationship may be alternative factors supporting a causal association. Indeed, evidences of reversibility of the effect and of a dose-response relationship were found in this study. A trend was identified for an increased risk for central obesity in current smokers and a reduced risk in former smokers. Moreover, among current smokers, a positive association was observed between smoking amount and central obesity. These results may imply a causal explanation.

The mechanisms accountable for these results have not been elucidated definitively. Various causal mechanisms may account for the effect of smoking on central obesity. First, smoking stimulates the sympathetic nervous system [15], leading to an increase of cortisol, a stress hormone [16], and abdominal fat deposition appears to be related to elevated levels of serum cortisol [17,18]. Second, cigarette smoking is related with increased insulin resistance [19,20], which is known to be associated with increases in abdominal fat deposition and diabetes [21,22]. Third, smoking has an anti-estrogenic effect [23]. This leads to an imbalance of sex hormones in both males and females, resulting in changes in fat metabolism and increased fat accumulation [24,25]. Finally, another possible reason is that smokers have distinct lifestyle characteristics, such as low intake of fruits and vegetables, a higher likelihood of depressive moods, and sleep impairments, which may increase their risk of becoming centrally obese [26,27].

This study has several limitations. First, no objective validation of smoking was performed using biochemical assays such as urine cotinine. The possibility of recall bias exists because information was drawn from the subjects’ recollection of their own behavior. Second, selection bias may have been present in the study. After excluding subjects with missing data, only 5,254 participants (21.6%) were included in this study. The participants who were included may have been different from those with missing or incomplete data. Third, the period of smoking in years and the total lifetime consumption of cigarettes in terms of pack-years were not included in the data. A precise dose-dependent relationship between smoking amount and obesity could therefore not be evaluated. Fourth, since this was a cross-sectional study, a cause-and-effect relationship cannot be established. Association does not imply causation. Finally, although we attempted to adjust for confounding factors, the possible impact of residual confounding factors, such as stress, sleep, mood, educational level, energy intake, and secondhand smoking, cannot be completely ruled out [26-28].

Despite these limitations, the main strength of this study is that it used the large dataset of the KNHANES, which is representative of the entire Korean population. The KHNANES collected a wide range of variables, including assessments of alcohol intake, physical activity, and the presence of hypertension and diabetes. Therefore, potential confounders could be adjusted for. The use of anthropometric measurements rather than self-reported weight, height, and WC is also one of the strengths of this study. People have a tendency to over-report height and to under-report body weight, thereby resulting in an misestimation of BMI [29]. Using self-reported anthropometric measurements to define obesity may result in overestimating the association between any health behavior and obesity.

In conclusion, this study found that current smokers are more likely to be centrally obese. A cause-and-effect relationship between smoking and central obesity cannot be conclusively identified due to the cross-sectional design of our study and the limitations mentioned above. However, current smokers should be enlightened that they may be more susceptible to central obesity.

## Figures and Tables

**Table 1.** General characteristics of the subjects

	Smoking status			p-value^1^
	Never	Current	Former	
Age (yr)	44.64±0.45	43.13±0.53	51.59±0.61	< 0.001
Height (cm)	160.92±0.24	169.69±0.27	167.90±0.27	< 0.001
Weight (kg)	60.81 ±0.29	69.79±0.39	67.91 ±0.36	< 0.001
BMI (kg/m^2^)	23.45 ±0.10	24.06 ±0.11	24.04±0.11	< 0.001
WC (cm)	78.42 ±0.27	83.24±0.35	83.96±0.33	< 0.001
Sex				< 0.001
Male	19.2 (1.0)	38.4 (1.1)	42.4 (1.1)	
Female	86.9 (1.0)	7.1 (1.1)	6.0 (1.1)	
Alcohol consumption (times/wk)				< 0.001
<1	64.2 (0.8)	11.3 (0.8)	24.5 (1.2)	
1	62.3 (1.0)	17.5 (2.0)	20.2 (1.6)	
2-3	31.2 (0.6)	40.6 (1.7)	28.3 (1.2)	
≥4	19.5 (0.3)	45.1 (1.1)	35.4 (1.0)	
Physical activity				< 0.001
Low	56.6 (1.1)	51.6 (1.7)	47.1 (1.8)	
Moderate	27.7 (1.0)	24.4 (1.3)	29.9 (1.4)	
High	15.7 (0.9)	24.0 (1.6)	23.0 (1.5)	
Hypertension				< 0.001
No	75.1 (0.9)	77.5 (1.2)	57.0 (0.8)	
Yes	24.9 (0.9)	22.5 (1.2)	43.0 (1.7)	
Diabetes				< 0.001
No	85.2 (0.6)	81.8 (1.1)	70.9 (1.2)	
Yes	14.8 (0.6)	18.2 (1.1)	29.1 (1.2)	

Values are presented as mean±SE or proportions (SE). BMI, body mass index; WC, waist circumference; SE, standard error.

1 Chi-square test for categorical variables, analysis of variance for continuous variables.

**Table 2.** Differences in BMI and WC according to smoking status^1^

	BMI		WC	
	Crude	Adjusted^2^	Crude	Adjusted^2^
Smoking status				
Never	23.45 (23.26, 23.64)	24.40 (24.10, 24.69)	78.42 (77.88, 78.96)	82.76 (82.04, 83.47)
Current	24.16 (24.95, 24.37)	24.44 (24.15, 24.74)	83.24 (82.56, 83.93)	83.68 (82.89, 84.47)
Former	24.04 (23.83, 24.25)	23.97 (23.68, 24.27)	83.96 (83.32, 84.60)	82.75 (81.96, 83.53)
p-value	< 0.001	0.005	< 0.001	0.06

Values are presented as mean (95% confidence interval) BMI, body mass index; WC, waist circumference.

1 Data were analyzed using analysis of covariance.

2 Adjusted for age, sex, physical activity, alcohol consumption, hypertension, and diabetes.

**Table 3.** Distribution of obesity by smoking status

	Obese		Centrally obese	
	Yes	No	Yes	No
Smoking status				
Never	69.2 (1.4)	30.8 (0.9)	76.9 (1.6)	23.1 (0.9)
Current	63.7 (1.4)	37.3 (1.0)	72.8 (1.7)	27.2 (1.0)
Former	32.1 (1.2)	37.9 (0.7)	70.1 (1.5)	29.9 (0.7)
p-value^1^	<0.001		<0.001	

Values are presented as proportions (standard error).

1 p-values are calculated by chi-square test.

**Table 4.** Risk of obesity by smoking status, compared to never-smokers^1^

	Obesity		Central obesity	
	Crude	Adjusted^2^	Crude	Adjusted^2^
Smoking status				
Never	1.00 (reference)	1.00 (reference)	1.00 (reference)	1.00 (reference)
Current	1.50 (1.27, 1.77)	1.14 (0.92, 1.41)	1.37 (1.14, 1.65)	1.30 (1.02, 1.67)
Former	1.36 (1.16, 1.61)	0.88 (0.70, 1.10)	1.37 (1.15, 1.63)	0.95 (0.74, 1.22)
p for trend	< 0.001	0.06	<0.001	0.02

Values are presented as odds ratio (95% confidence interval).

1 Data were analyzed using multiple logistic regression analysis.

2 Adjusted for age, sex, physical activity, alcohol consumption, hypertension, and diabetes.

**Table 5.** Risk of obesity by daily smoking amount in current smokers, compared to never-smokers^1^

	Obesity		Central obesity	
	Crude	Adjusted^2^	Crude	Adjusted^2^
Smoking amount (cigarette/d)				
1-10	1.00 (reference)	1.00 (reference)	1.00 (reference)	1.00 (reference)
11-20	1.27 (0.94, 1.70)	1.20 (0.89, 1.60)	1.04 (0.74, 1.45)	1.03 (0.73, 1.45)
> 20	1.43 (0.79, 2.58)	1.45 (0.80, 2.63)	1.52 (0.84, 2.76)	1.41 (0.79, 2.53)
p for trend	0.25	0.34	0.35	0.49

Values are presented as odds ratio (95% confidence interval).

1 Data were analyzed using multiple logistic regression analysis.

2 Adjusted for age, sex, physical activity, alcohol consumption, hypertension, and diabetes.
