# Supplementary material for: Associations of smoking with overall obesity, and central obesity: a cross-sectional study from the Korea National Health and Nutrition Examination Survey (2010-2013)
Source: Epidemiol Health. 2016 May 19;38:e2016020. doi: 10.4178/epih.e2016020 (PMC4967909; doi:10.4178/epih.e2016020)
Supplement: Supplementary file 1 [file epih-38-e2016020-app.pdf]

Appendix 1. Differences of BMI and WC according to smoking amount of current smokers[1]

	BMI		WC	
	Crude	Adjusted[2]	Crude	Adjusted[2]
Smoking amount (cigarette/d)				
1-10	23.83 (23.46, 24.20)	23.84 (23.29, 24.40)	81.85 (80.70, 83.01)	82.12 (80.62, 83.62)
11-20	24.36 (24.05, 24.66)	24.16 (23.56, 24.76)	83.96 (83.10, 84.82)	82.69 (81.15, 83.23)
>20	24.58 (23.79, 25.37)	24.50 (23.66, 25.33)	85.93 (83.72, 88.14)	84.18 (81.90, 86.45)
p-value	0.09	0.30	0.001	0.23

Values are presented as mean (95% confidence interval).
BMI, body mass index; WC, waist circumference.
[1]Data were analyzed using analysis of covariance.
[2]Adjusted for age, sex, physical activity, alcohol consumption, hypertension, and diabetes.

Appendix 2. Obesity distribution of current smokers by daily smoking amount

	Obese		Centrally obese	
	Yes	No	Yes	No
Smoking amount (cigarette/d)				
1-10	33.8 (2.1)	66.2 (2.7)	25.7 (1.9)	74.3 (3.3)
11-20	39.3 (2.1)	60.7 (2.6)	27.7 (2.0)	72.3 (3.2)
>20	44.6 (1.1)	55.4 (1.5)	33.7 (1.0)	66.3 (2.0)
p-value[1]	0.21		0.35	

Values are presented as proportions (standard error).
[1]p-values are calculated by chi-square test.
